# Identification of gene biomarkers with expression profiles in patients with allergic rhinitis

**DOI:** 10.1186/s13223-022-00656-4

**Published:** 2022-03-04

**Authors:** Yun Hao, Boqian Wang, Jinming Zhao, Ping Wang, Yali Zhao, Xiangdong Wang, Yan Zhao, Luo Zhang

**Affiliations:** 1 Department of Otolaryngology Head and Neck Surgery and Department of Allergy, Beijing TongRen Hospital, Capital Medical University, Beijing, 100730 China; 2 Beijing Laboratory of Allergic Diseases and Beijing Key Laboratory of Nasal Diseases, Beijing Institute of Otolaryngology, Beijing, 100005 China; 3 Research Unit of Diagnosis and Treatment of Chronic Nasal Diseases, Chinese Academy of Medical Sciences, Beijing, 100005 China

**Keywords:** Allergic rhinitis, Differentially expressed genes, Bioinformatic analysis, Biomarkers, Diagnosis

## Abstract

**Background:**

Allergic rhinitis (AR) is an upper respiratory tract inflammation disease caused by IgE-mediated reactions against inhaled allergens. The incidence of AR is significantly increasing throughout the world. Hence, more specific, and sensitive gene biomarkers and understanding the underlying pathways are necessary to further explore the AR pathogenesis.

**Objective:**

To identify gene biomarkers in nasal mucosa and in blood from AR patients which could be used in AR diagnosis.

**Methods:**

The gene expression profiles of GSE43523 from nasal epithelial cells and GSE75011 from Th2-enriched CD4+ T cells in blood were downloaded from the Gene Expression Omnibus database. Gene Ontology (GO), Kyoto Encyclopedia of Genes and Genomes (KEGG) analyses and protein–protein interaction (PPI) network analysis were conducted to investigate the functional changes of genes. The receiver operating characteristic (ROC) curves were used to assess the diagnostic values of the hub genes. Real-time quantitative PCR (RT-qPCR) was performed to validate the hub genes.

**Results:**

Significant differentially enriched gene signatures in AR patients were identified in nasal epithelial cells (n-DEGs) and in blood (t-DEGs). Signatures associated with axoneme, extracellular matrix, collagen fibril organization, cell motility, calcium ion binding, and so on were more enriched in n-DEGs, whereas signatures associated with TNF signaling pathway, detoxification of inorganic compound, and cellular response to corticotropin-releasing hormone stimulus were enriched in t-DEGs. In addition, we identified 8 hub genes and 14 hub genes from n-DEGs and t-DEGs, respectively. The combination of *POSTN* in nasal mucosa and *PENK* and *CDC25A* in blood was constructed with a good AR predicting performance. The area under the curve (AUC) of the ROC curve of 3 hub genes’ combination was 0.98 for AR diagnosis.

**Conclusion:**

This study utilized gene expression profiles and RT-qPCR validation on nasal mucosa and blood from AR patients to investigate the potential biomarkers for AR diagnosis.

**Supplementary Information:**

The online version contains supplementary material available at 10.1186/s13223-022-00656-4.

## Background

Allergic rhinitis (AR) is defined as symptoms of nasal obstruction, rhinorrhea, sneezing, and nasal pruritus caused by IgE-mediated reactions against inhaled allergens and involving mucosal inflammation driven by type 2 helper T (Th2) cells [1]. It affects approximately 17 to 28.5% of adults in Europe and 10 to 30% of adults in the United States [2]. It has been previously shown that the prevalence of AR has increased from 11.1 to 17.6% in adults in China [3]. AR is also associated with comorbidities such as asthma, rhinosinusitis, conjunctivitis, nasal polyposis and otitis media [4]. Recent studies have demonstrated that an imbalance in innate and adaptive immunity together with environmental factors plays a key role in AR pathogenesis [5]. Serum IgE in AR may be derived from nasal mucosal production [6], suggesting that research on nasal mucosa and peripheral blood in AR patients should not be separated. Although significant advances have been made in understanding the pathophysiology of AR, its early diagnosis, therapeutic intervention, and underlying pathogenesis remain challenges. Therefore, elucidating the unique characteristics of AR is important for developing biomarkers for its diagnosis and novel clinical strategies for its treatment.

Currently, next-generation sequencing and bioinformatic analysis have emerged as promising, useful tools for screening genetic alterations involved in the development and progression of diseases, with marked clinical applications ranging from molecular diagnosis to disease classification, patient stratification to therapeutic prediction, and new drug target discovery to response prediction [7, 8]. Previous studies have utilized samples from nasal epithelial cells, peripheral blood mononuclear cells (PBMCs) or Th2-enriched CD4+ T cells to identify differentially expressed genes (DEGs) in AR patients compared to healthy controls [9–11]. Although some studies have focused on the analysis of AR-related DEGs, there is a lack of an integrative analysis of local and circulating samples to elucidate the complexity of AR pathogenesis.

The utility of AR biomarkers for clinical trials depends on both sensitivity of the measure but also the practicality of the testing and sampling. Both the collection of nasal epithelial cells and peripheral blood are rapid and minimally invasive procedures. Real-time quantitative PCR (RT-qPCR), as one of the most financially accessible and widely available detection technologies is commonly used for the identification of gene biomarkers both in clinical and research studies.

In this study, we aimed to explore the possible molecular mechanisms by integrating databases on nasal epithelial cells and peripheral Th2-enriched CD4+ cells from AR patients and the same cells from healthy controls. We described the transcriptional features and identified gene biomarkers to shed light on AR development at the molecular level and to pave the way toward understanding potential pathogenesis mechanisms to facilitate its diagnosis.

## Methods

### Ethics approval and consent to participate

The study was approved by the Ethics Committee of Beijing TongRen Hospital, Capital Medical University in accordance with the guidelines of the World Medical Association’s Declaration of Helsinki., and informed consent was obtained from all participants before the study.

A total of 20 subjects, including 10 AR patients and 10 healthy controls were recruited in this study. The diagnosis of AR was made according to the Allergic Rhinitis and its Impact on Asthma (ARIA) guidelines [12], including (1) presence of persistent or discontinuous symptoms of continuous sneezing, anterior rhinorrhoea, nasal obstruction, and itching, (2) a pale and edematous nasal mucosa, nasal discharge, and swollen inferior turbinate by nasal endoscopy, (3) positive antigen sIgE in serum that measured by the ImmunoCAP 1000 system (Pharmacia, Uppsala, Sweden). The result of the serum specific IgE consists of D1, D2 and PHAD. D1 stands for *Dermatophagoides pteronyssinus*, D2 stands for *Dermatophagoides farinae*, and PHAD stands for phadiatop, which suggests a respiratory allergy to inhalant allergens. Subjects were considered “positive” for AR diagnosis more than a cutoff of 0.35 kU/L of serum sIgE. The exclusion criteria are including: (1) co-morbid asthma, eczema, or any other allergic diseases; (2) hypertension, diabetes, or other chronic diseases; or (3) tumor in the nasal cavity or any other inflammatory nasal disease. Details of the subjects’ characteristics are included in Table 1.

**Table 1:** Clinical characteristics of healthy controls and AR patients

	AR (n = 10)	Control (n = 10)	*P* value
Age (year, mean ± SD)	34.3 ± 1.461	31.6 ± 3.121	0.4435
Sex (Female/Male)	6/4	6/4	> 0.9999
VAS (mean ± SD)	5.475 ± 0.7139	0	< 0.0001
PHAD (KUA/L, mean ± SD)	9.096 ± 3.78	0.181 ± 0.02519	0.0299
D1 (KUA/L, mean ± SD)	3.412 ± 1.963	0.17 ± 0.02556	0.116
D2 (KUA/L, mean ± SD)	7.115 ± 4.249	0.148 ± 0.02375	0.1184
Total IgE (KUA/L, mean ± SD)	383.8 ± 142	23.16 ± 2.395	0.0205

*AR* allergic rhinitis, *VAS* visual analog scale, *SD* standard deviation, *PHAD* phadiatop

All subjects underwent nasal brushing of the 1 nare. The brush samples were immediately put into phosphate buffered saline containing 2% bovine serum albumin on ice, underwent RNA isolation after centrifugate at 300*g*, 10 min, at 4 ℃. The 2 mL whole blood samples were collected with tubes with EDTA anticoagulant, then lysis by 10× red blood cell lysis buffer and then washed twice by phosphate buffered saline. Total RNA was extracted from nasal brush samples and blood samples according to the manufacturer’s protocol of TRIzol reagent (Sigma-Aldrich) for real-time quantitative PCR analysis.

### Microarrays, datasets, and characteristics of clinical samples from the Gene Expression Omnibus (GEO) data repository

GSE43523 (https://www.ncbi.nlm.nih.gov/geo/query/acc.cgi?acc=GSE43523), a dataset derived from nasal epithelial cells, and GSE75011 (https://www.ncbi.nlm.nih.gov/geo/query/acc.cgi?acc=GSE75011), a dataset derived from Th2-enriched CD4+ T cells from peripheral blood, were obtained from the GEO database. GSE43523, which includes seven samples from patients with seasonal AR (SAR) and five samples from non‑allergic healthy controls, was derived from the GPL6883 platform (Illumina HumanRef-8 v3.0 expression bead chip), and GSE75011, which includes 25 samples from patients with AR and 15 samples from healthy controls, was derived from the GPL16791 platform (Illumina HiSeq 2500) [11]. The flow chart of our study is shown in Additional file 1: Figure S1.

### Differential gene expression analysis

First, background correction and standardization were performed on the original GEO datasets using the packages edgeR and limma of R software [13]. Next, differential gene analysis (|log_2_FC|> 1, *P*-value < 0.05) was performed to compare AR patients and healthy controls with the limma package of R software. Heatmaps and volcano plots of the differentially expressed genes (DEGs) were constructed using the packages pheatmap and ggplot2 of R software [14].

### Functional enrichment and pathway analyses

We analyzed the functions and signaling pathways of the DEGs using MetaCore™ (version 20.3, Thomson Reuters, New York, NY, USA), a computational platform capable of analyzing gene clusters in the context of functional enrichment and pathway analyses. Pathway maps and Gene Ontology (GO) analyses were performed in MetaCore [15]. GO annotation includes three kinds of functional categories: biological process (BP), cellular component (CC) and molecular function (MF). Functional enrichment analysis was based on the cutoff value of *P* < 0.05.

### Protein–protein interaction (PPI) network construction

STRING (version 11.0) (http://string-db.org/) was used to identify the PPIs of the intersecting DEGs of the two datasets, with a combined score > 0.4 used as the threshold for statistically significant interactions [16]. Cytoscape (version 3.7.2) software was used to further visualize the PPI network [17]. Then, the ClueGO and CluePedia plugins were used to decipher functionally grouped GO and pathway annotation networks in the PPI network [18]. A *P*-value < 0.05 and a κ coefficient of 0.4 were considered the threshold values. Then, the Molecular Complex Detection (MCODE) plugin was used to identify important proteins in the PPI network [19]. In addition, the degree cutoff was set as 2, the node score was set as 0.2, the k-score was set as 2, and the maximum depth was set as 100. Proteins with a false discovery rate (FDR) < 0.05 were considered statistically significant. The ClueGO plugin was then used to perform GO and Kyoto Encyclopedia of Genes and Genomes (KEGG) functional enrichment analyses. Kappa coefficients were calculated to reflect the functional correlations between GO terms or KEGG pathways and were defaulted to values greater than or equal to 0.4 [20].

### Circular visualization

A circular layout is a convenient and efficient way to visualize large amounts of genomic information. The circlize package of R software was utilized to visualize relations among the chromosome locations, gene biotypes, and expression levels of the DEGs [21].

### Reverse transcription and real-time quantitative PCR (RT-qPCR)

Total RNA was isolated from lysates of nasal brushing cells and white blood cells using TRIzol reagent (Sigma-Aldrich) according to the manufacturer’s protocol. The concentration and purity of the RNA was assessed with a Nanodrop-2000 (Thermo Fisher Scientific). RNase-free water was used to adjust the stock concentration of all RNA samples to 500 ng/μL. Complementary DNA was synthesized from 1.5 μg of total RNA using a High-Capacity cDNA Reverse Transcription Kit with genomic DNA remover (Abclonal Biotechnology, #RK20403). In order to avoid the genomic DNA contamination, total RNA was treated with 4× gDNA remover mix t for 5 min at 37 °C, then for additional 5 min at 85 °C, and stop at 4 °C in a 20 μL reaction system. Then, total RNA with genomic DNA removal was treated with 5× RT mix for 5 min at 25 °C, for 15 min at 42 °C, for 5 s at 85 °C, and stop at 4 °C. All RT-qPCR products were amplified using a SYBR Green I Master Kit (Abclonal Biotechnology, #RK21202) in 10 µL reaction volume on an Applied Biosystems^®^ 7500 Real-Time PCR System. The reaction mix contains 5 µL of 2× SYBR Green Fast mix, 3 µL of RNase-free water and 0.5 µL each of forward and reverse primer and 1 µL of cDNA. The negative control for RT-qPCR used RNase-free water instead of cDNA template. The RT-qPCR reaction conditions were 95 °C 2 min followed by 40 cycles of 95 °C for 15 s, 55 °C for 15 s and 72 °C 30 s. Then, the last cycle was 95 °C for 15 s, 60 °C for 15 s and 95 °C for 15 s for melt curve analysis. Each reaction had three replicates. The RT-qPCR products were confirmed by sequencing. All primers in the negative control group did not have any amplification. Expression levels were normalized to human glyceraldehyde 3-phosphate dehydrogenase gene (*GAPDH*). The relative gene expression level is for target genes relative to reference gene. The gene expression level was calculated as 2(^−△△Ct^) method, and the △Ct means the Ct of target gene minus the Ct of reference gene. The *GAPDH* forward primer was 5ʹ-CGGAGTCAACGGATTTGGTC-3ʹ, and *GAPDH* reverse primer was 5ʹ-TGGGTGGAATCATATTGGAACAT-3ʹ. The *POSTN* forward primer was 5ʹ-TGCCCAGCAGTTTTGCCCAT-3ʹ, and *POSTN* reverse primer was 5ʹ-CGTTGCTCTCCAAACCTCTA-3ʹ. The *PENK* forward primer was 5ʹ-TAATGGAATGTGAAGGTAA-3ʹ, and *PENK* reverse primer was 5ʹ-GGTTTGCTATTTTCTCTG-3ʹ. The *CDC25A* forward primer was 5ʹ-TGGAGGTGAAGAACAACA-3ʹ, and *CDC25A* reverse primer was 5ʹ-GGTCAAGAGAATCAGAATGG-3ʹ.

### Statistical analysis

A receiver operating characteristic (ROC) curve was drawn to calculate the area under the curve (AUC) to discriminate AR patients from normal controls in all AR GEO datasets. SPSS 16.0 for Windows (IBM, Chicago, USA) was used for ROC curve analyses, and other statistical analyses were performed using GraphPad Prism 7.0 software (GraphPad Software, La Jolla, CA).

## Results

### Identification of DEGs in AR

We performed a differential analysis of mRNAs and identified 997 DEGs in nasal epithelial cells from AR patients (n-DEGs), of which 559 were down-regulated and 438 were up-regulated (|log_2_FC|> 1, adjusted *P*-value < 0.05). The same method was used to analyze and identify 165 DEGs (78 up-regulated and 87 down-regulated) in Th2-enriched CD4+ T cells from peripheral blood samples (t-DEGs) in GSE75011 using the edge and limma packages in R.

**Fig. 1:**
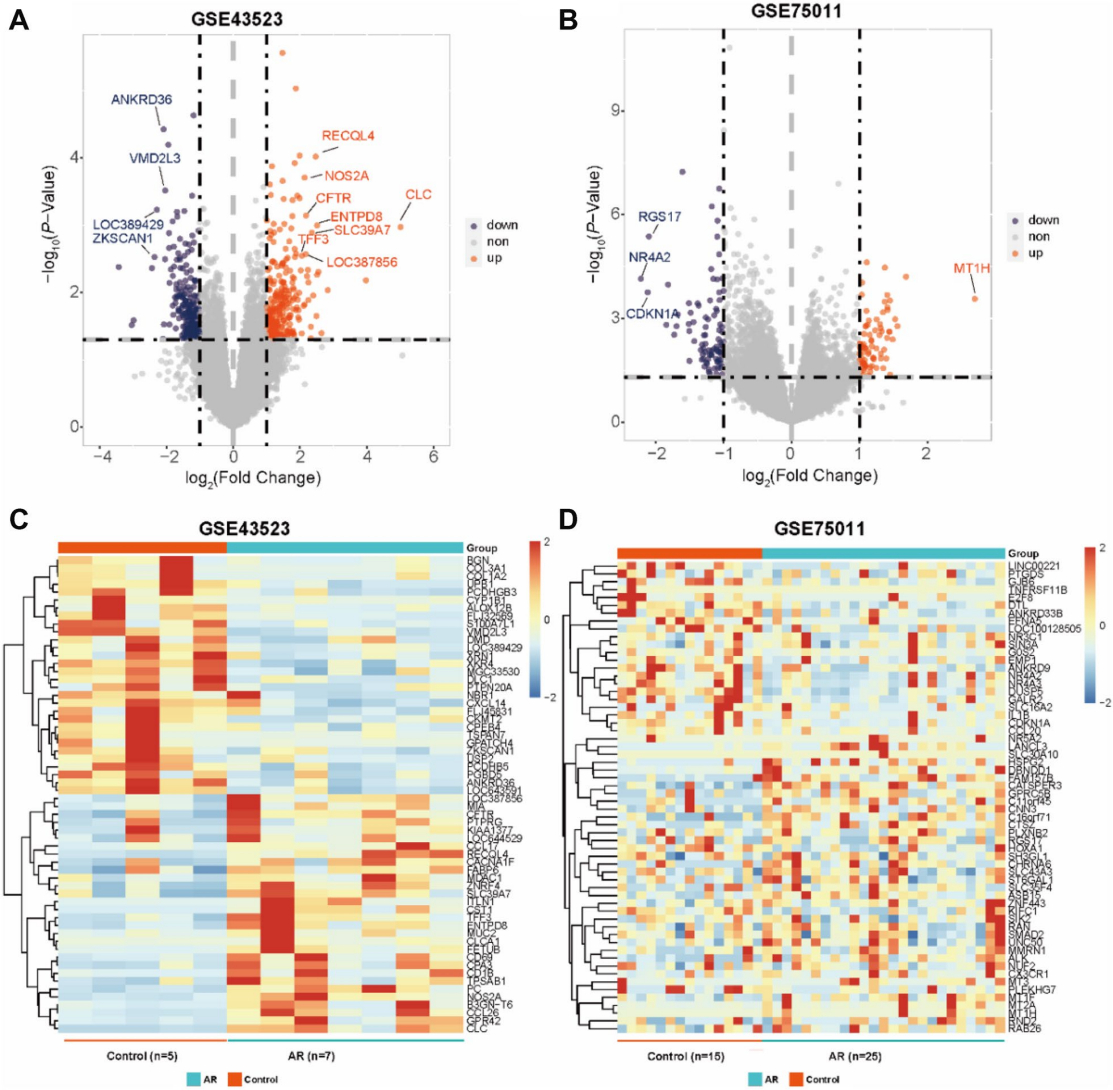
Identification of differentially expressed genes (DEGs) in AR. **A**, **B** Volcano plots of **A** n-DEGs and **B** t-DEGs. **C**, **D** Heatmaps of **C** n-DEGs and **D** t-DEGs. Genes with a |log_2_FC|> 1 and a *P*-value < 0.05 are shown in volcano plots. Orange represents up-regulated genes in AR, blue represents down-regulated genes in AR, and gray represents no significant change. n-DEGs, differentially expressed genes in the nasal epithelial cells of AR samples, t-DEGs, differentially expressed genes in Th2-enriched CD4+ T cells from peripheral blood samples

Volcano plots were used to display the gene expression data and *P*-value statistics of each dataset (Fig. 1A, B). We identified the top 50 genes that were significantly differentially expressed (|log_2_FC|> 1, adjusted *P*-value < 0.05) between AR patients and healthy controls in the heatmap (Fig. 1C, D).

### Pathway maps and GO functional enrichment in AR by MetaCore

**Fig. 2:**
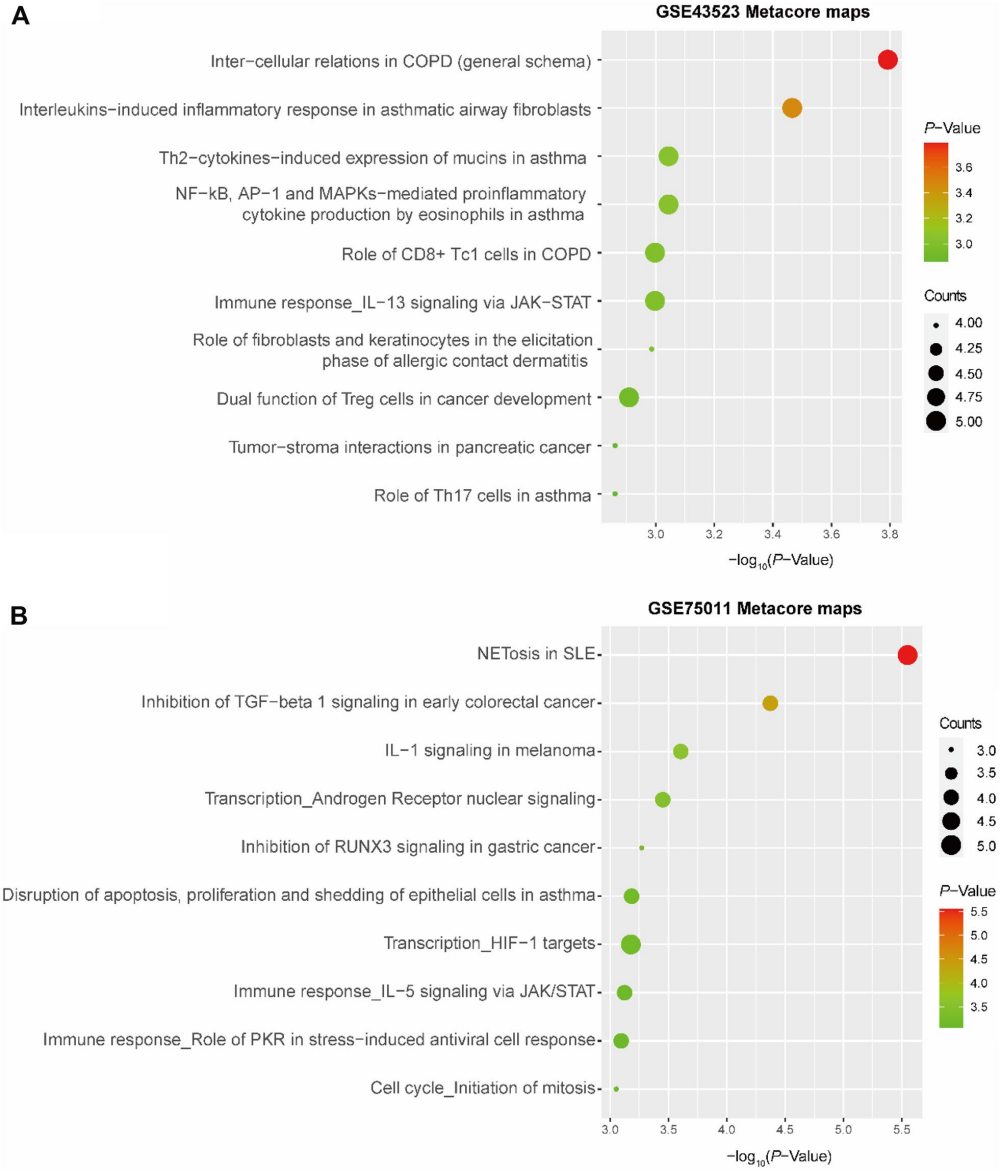
Pathway maps of DEGs in AR. **A**, **B** Bubble chart showing pathway maps for **A** n-DEGs and **B** t-DEGs by MetaCore

To explore the potential functions of the DEGs, we generated pathway maps and performed GO functional enrichment analysis in MetaCore (*P*-value < 0.05). The top ten significantly enriched pathways in AR (according to the pathway map) are shown in Fig. 2. The pathway map terms associated with epithelial cells in AR patients were related to chronic obstructive pulmonary disease (COPD) and asthma (Fig. 2A) (e.g., intercellular communication, the role of CD8+ Tc1 cells in COPD, the interleukin-induced inflammatory response in asthmatic airway fibroblasts, the Th2 cytokine-induced expression of mucins, NF-κB AP-1 and MAPK-mediated proinflammatory cytokine production by eosinophils and the role of Th17 cells in asthma). On the other hand, NETosis in systemic lupus erythematosus (SLE), inhibition of TGF-β1 signaling, IL-1 signaling, transcription androgen receptor nuclear signaling, and inhibition of RUNX3 signaling were the main enriched pathways in Th2-enriched CD4+ T cells from peripheral blood in AR patients (Fig. 2B).

**Fig. 3:**
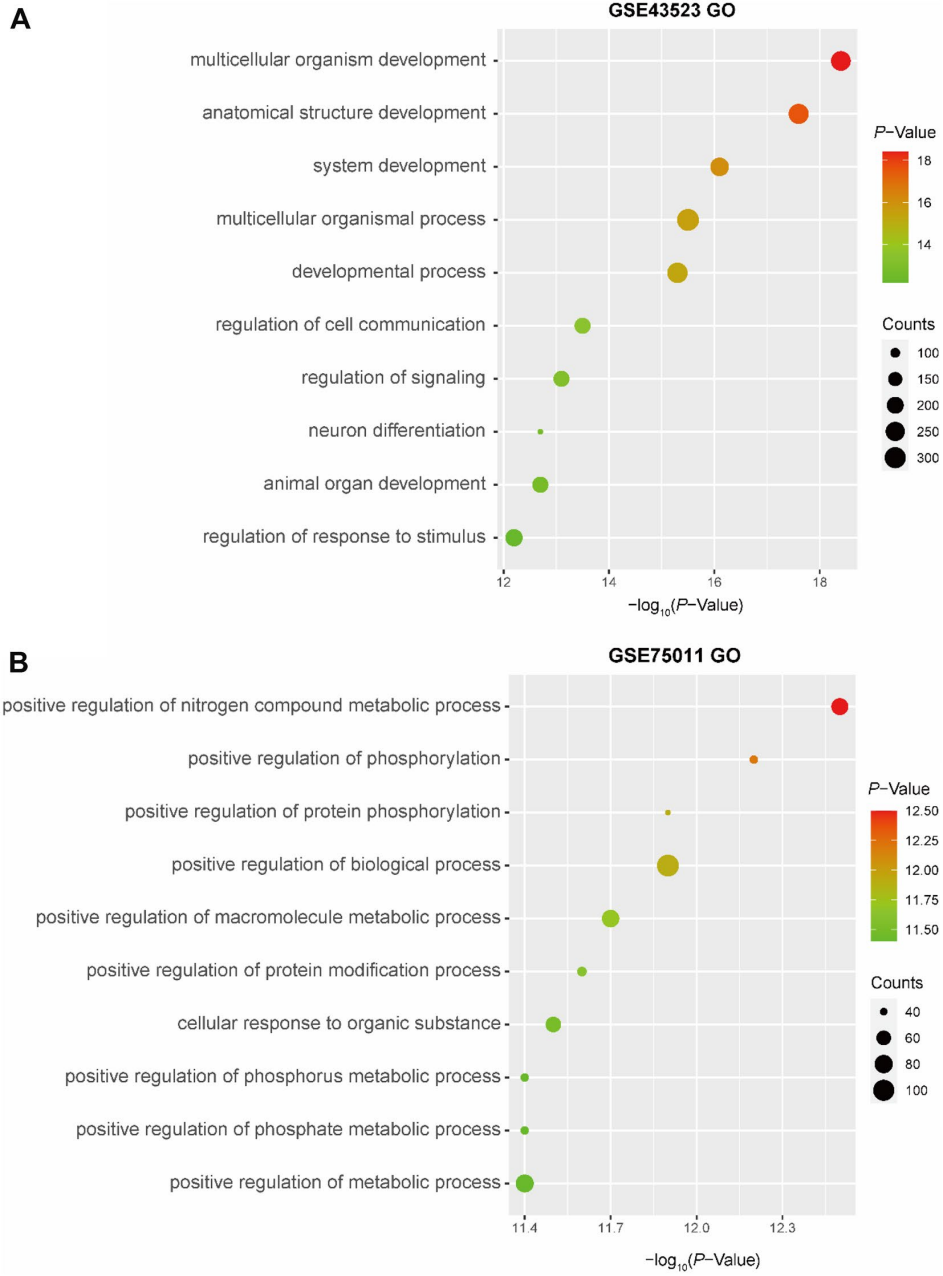
GO functional enrichment of DEGs in AR. **A**, **B** GO functional enrichment for **A** n-DEGs and **B** t-DEGs by MetaCore

The n-DEGs in AR patients were also strongly associated with the GO BP terms multicellular organism development, anatomical structure development, and system development process (Fig. 3A), while t-DEGs in AR patients were mainly related to positive regulation of nitrogen compound metabolic process, positive regulation of phosphorylation, and positive regulation of protein phosphorylation (Fig. 3B).

### PPI networks and hub DEGs in AR

**Fig. 4:**
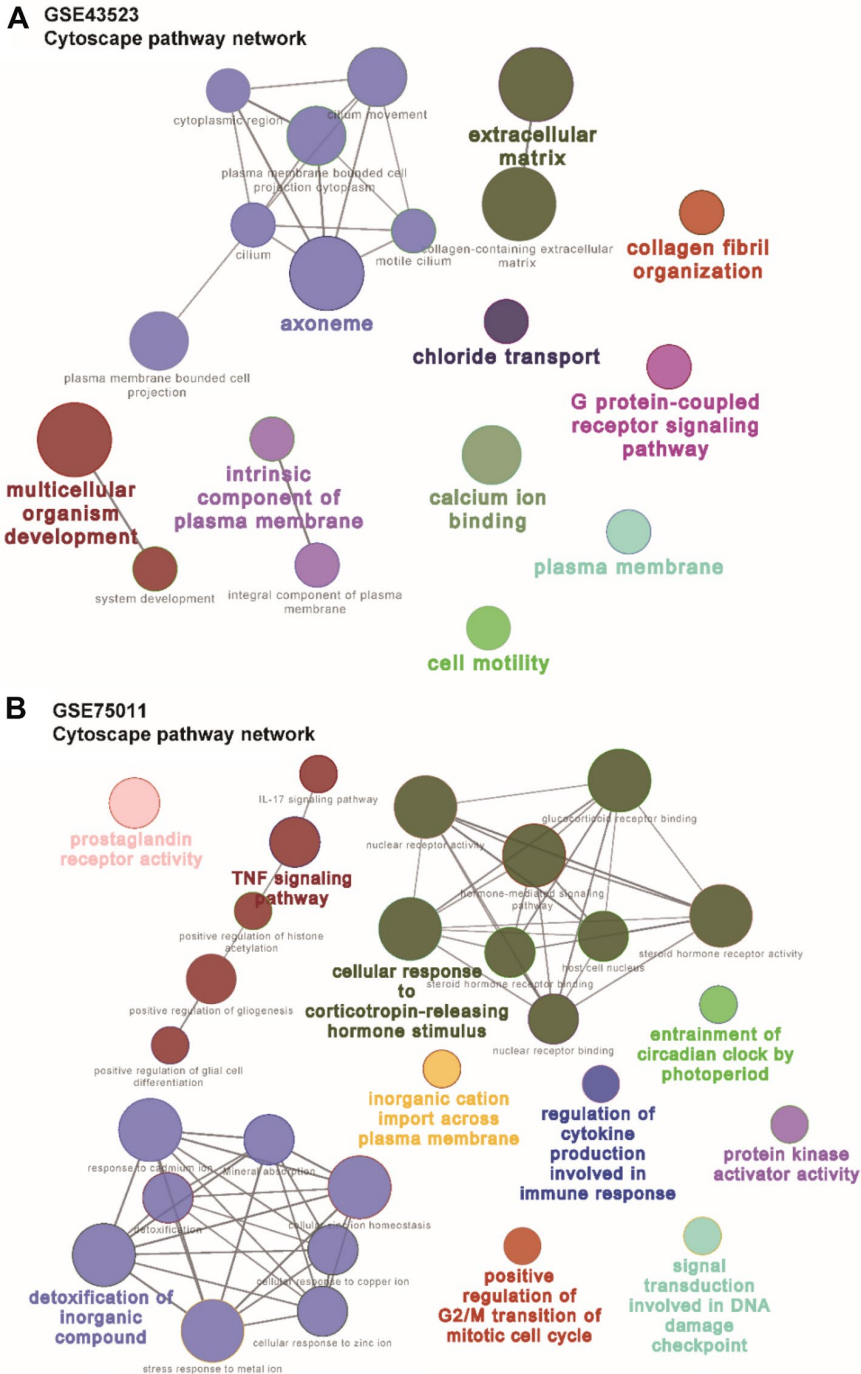
PPI networks of DEGs in AR. **A**, **B** PPI networks for **A** n-DEGs and **B** t-DEGs. The node color represents the degree of proteins, and the edge color represents the combined score of proteins. Orange represents high, and blue represents low. **C**, **D** (**C**) n-DEGs and (**D**) t-DEGs in the PPI networks

To illuminate the relationships between the DEGs and pathways, the ClueGO plugin of Cytoscape was used to perform GO functional enrichment and KEGG pathway analyses. Each node and line represent a term and the correlation between the terms, respectively. The color of the term shows the classification of nodes according to the functions. The results indicated that the n-DEGs were significantly enriched in axoneme, extracellular matrix, collagen fibril organization, cell motility, calcium ion binding, and so on (Fig. 4A), while the t-DEGs were enriched in the TNF signaling pathway, detoxification of inorganic compound, and cellular response to corticotropin-releasing hormone stimulus (Fig. 4B).

**Fig. 5:**
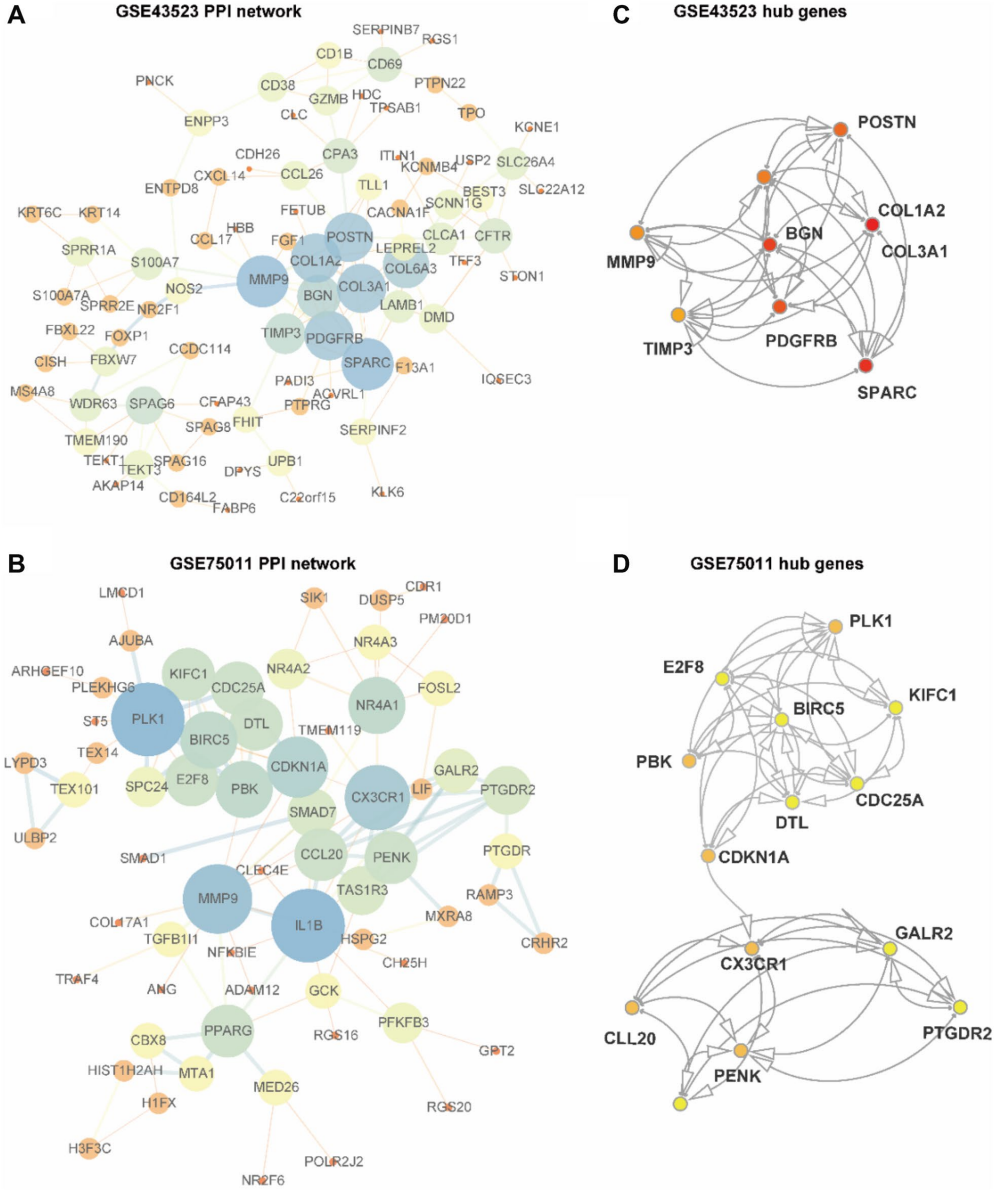
ClueGO for GO term and KEGG pathway analyses. **A**, **B** GO terms and KEGG pathways of **A** n-DEGs and **B** t-DEGs. Each node represents a GO term or KEGG pathway, and the size of the node is inversely associated with the P-value of enrichment. The color reflects the functional enrichment between the nodes, with the same function aggregating together in the same color. Genes with a kappa value > 0.4 are shown in the diagram

To further explore the connections among the DEGs, PPI networks were constructed with the n-DEGs and t-DEGs. The PPI networks of n-DEGs contained 119 nodes and 160 edges, and the isolated genes (without interactions) were removed. A total of 145 interactions and 94 nodes were screened to establish the PPI networks of t-DEGs (Fig. 5A, B). The MCODE algorithm was further applied to identify hub n-DEGs and t-DEGs that were densely associated with each other in the networks (Fig. 5C, D). We found 8 hub genes in the PPI networks of n-DEGs, namely, platelet-derived growth factor receptor β (*PDGFRB*), biglycan (*BGN*), secreted protein acidic and cysteine rich (*SPARC*), TIMP metallopeptidase inhibitor 3 (*TIMP3*), collagen type I α2 chain (*COL1A2*), collagen type III α1 chain (*COL3A1*), matrix metallopeptidase 9 (*MMP9*), and periostin (*POSTN*) and 14 hub t-DEGs, namely, E2F transcription factor 8 (*E2F8*), denticleless E3 ubiquitin protein ligase homolog (*DTL*), kinesin family member C1 (*KIFC1*), proenkephalin (*PENK*), galanin receptor 2 (*GALR2*), taste 1 receptor member 3 (*TAS1R3*), prostaglandin D2 receptor 2 (*PTGDR2*), C-X3-C motif chemokine receptor 1 (*CX3CR1*), C–C motif chemokine ligand 20 (*CCL20*), cyclin dependent kinase inhibitor 1A (*CDKN1A*), PDZ binding kinase (*PBK*), cell division cycle 25A (*CDC25A*), polo like kinase 1 (*PLK1*), and baculoviral IAP repeat containing 5 (*BIRC5*).

### Circular visualization of DEGs in AR

**Fig. 6:**
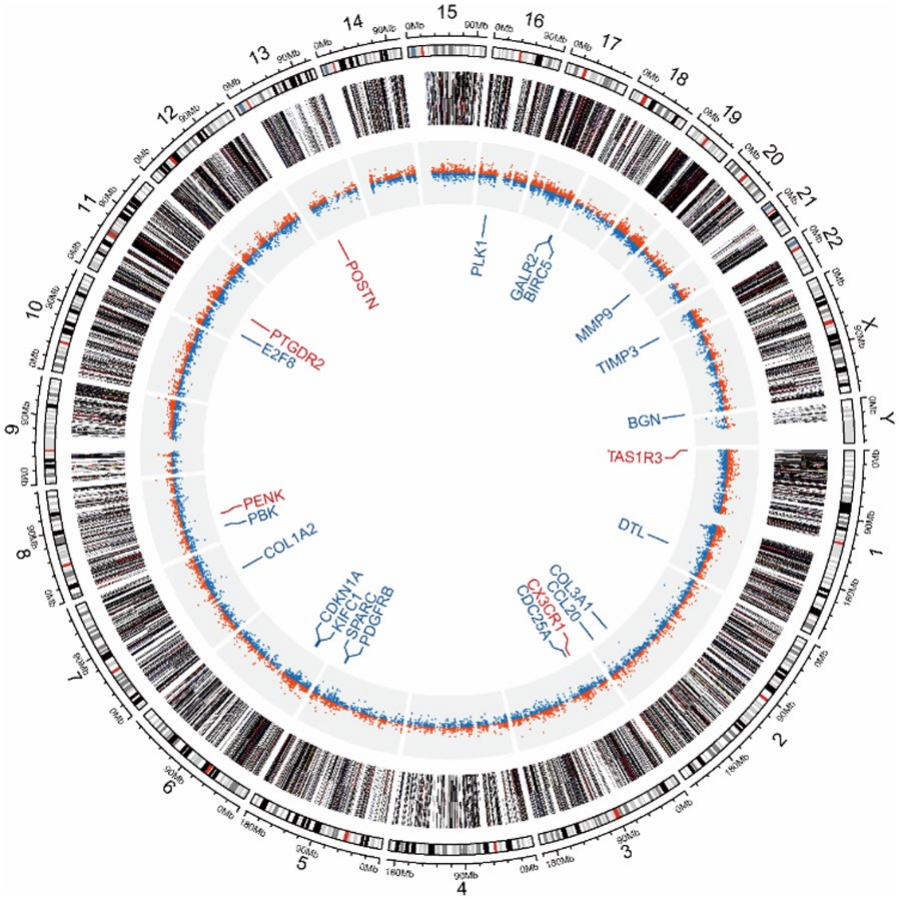
Circos plots representing the distribution of DEGs in AR on human chromosomes. The outermost track of the Circos plot is the chromosome map of the human genome. Track 2 represents different gene biotypes of all DEGs from GSE43523 and GSE75011. Black, gray, red, orange, navy, purple, green, blue, yellow represent protein coding, long noncoding RNA (lncRNA), microRNA (miRNA), miscellaneous RNA (miscRNA), pseudogene, small nucleolar RNA (snoRNA), small nuclear RNA (snRNA), to be experimentally confirmed RNA (TEC), and other RNA, respectively. Track 3 represents the degree of difference between AR patients and healthy controls. The up-regulated or down-regulated genes are marked with red or blue plots, respectively, and plot heights in track 3 indicate the log_2_FC value of all DEGs from GSE43523 and GSE75011. Track 4 shows the labels of hub genes from the two GEO datasets. Red represents up-regulated genes, and blue represents down-regulated genes

A circular layout is an advantageous approach to present information along several chromosomes, multidimensional descriptions on the same chromosome, and relations between genomic rearrangements simultaneously. To obtain a better view of the symbols, chromosome locations, and expression levels of the DEGs, we drew Circos plots with the circlize package (Fig. 6). In Circos, each concentric ring is called a ‘track’. First, the outermost track of the Circos plot was used to present the locations of chromosomes in the human genome assembly GRCh38 (hg38). Track 2 represents the different gene biotypes of all DEGs from the GSE43523 and GSE75011 datasets. Track 3 depicts the degree of difference between AR patients and healthy controls. The up-regulated or down-regulated genes are marked with red or blue plots, respectively, and plot heights in track 3 indicate the log_2_FC of all DEGs from the GSE43523 and GSE75011 datasets. To explore the locations of the hub genes from the two GEO datasets, track 4 was visualized, with red representing up-regulated genes and blue representing down-regulated genes. In general, chromosomes 2, 3, 5, 6, 8, 11, and 17 each contained 2 hub genes in AR. Different genes were proven to be relatively distributed on different chromosomes.

### Validation of hub genes based on GEO databases and RT-qPCR

**Fig. 7:**
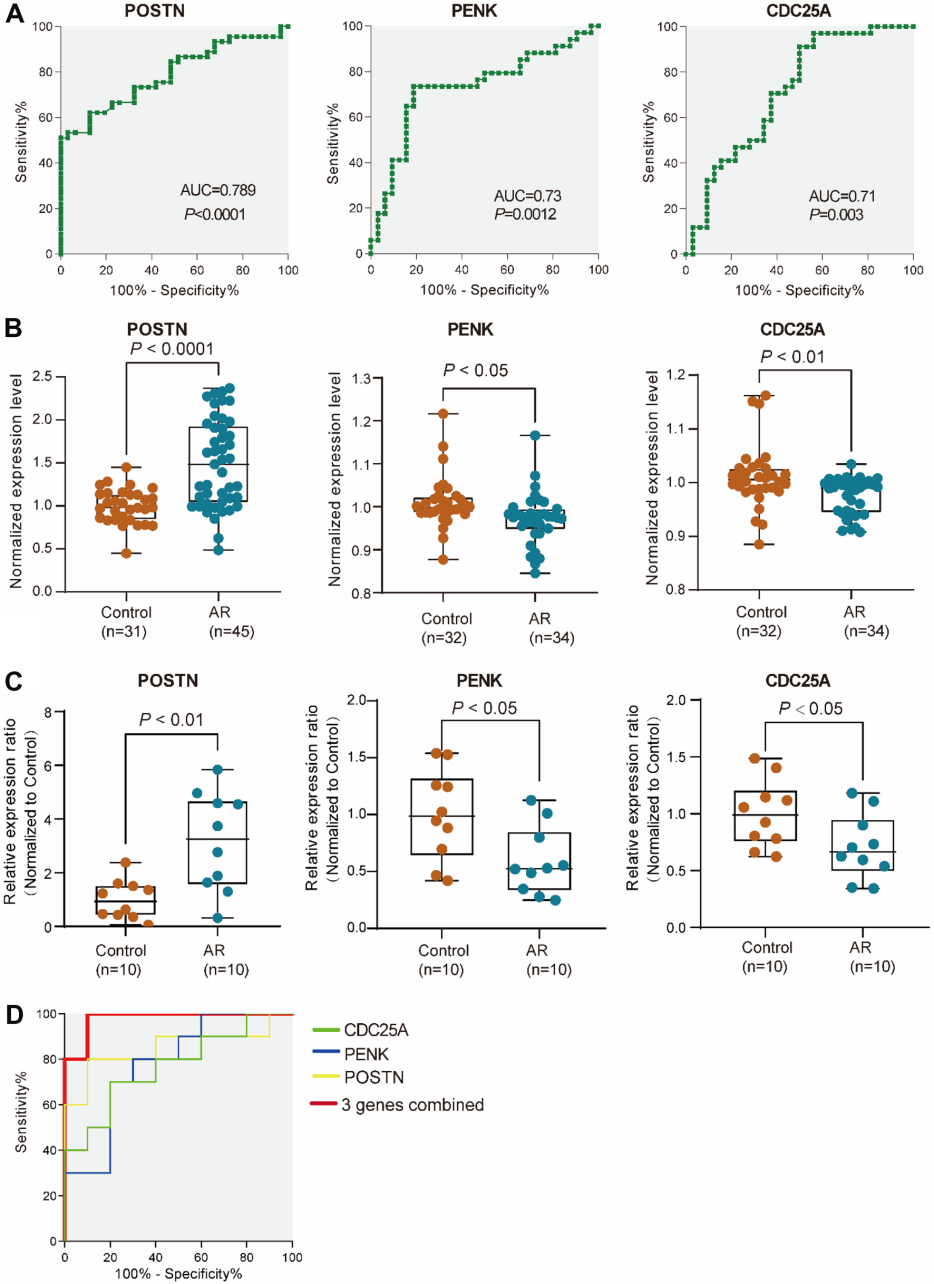
The diagnostic values and expression level of 3 hub genes in AR nasal mucosa and in blood, respectively. **A**, **B** ROC curves (**A**) and normalized relative expression levels (**B**) of *POSTN*, *PENK* and *CDC25A* in AR from public GEO datasets. The AUC of *POSTN*, *PENK* and *CDC25A* were 0.789, 0.73 and 0.71. n-DEGs from combination of GSE101720, GSE19187, GSE46171 and GSE44037 databases for *POSTN* analysis. And t-DEGs from GSE50101 and GSE37157 for *PENK* and *CDC25A* analysis. **C**, **D** The relative expression levels (**C**) and ROC curves (**D**) of *POSTN* in nasal mucosa and *PENK* and *CDC25A* in blood of AR patients. The AUC of combined detection of the 3 indexes was 0.98

To validate the correlations between these hub genes and AR, We compared the expression of each candidate hub gene in nasal samples based on GSE101720, GSE19187, GSE46171 and GSE44037. In addition, GSE50101 and GSE37157 were used for validation of hub genes in blood. The ROC analysis revealed that only the expression levels of *POSTN* in nasal mucosa and *PENK* and *CDC25A* in blood showed excellent diagnostic value for AR diagnosis (Fig. 7A, B). Furthermore, RT-qPCR was performed to analyze the expression levels of *POSTN* in nasal mucosa and *PENK* and *CDC25A* in blood from the AR patients. We found that the expression level of *POSTN* was significantly increased in nasal mucosa, while the expression levels of *PENK* and *CDC25A* were decreased in blood of AR patients (Fig. 7C and Additional file 1: Tables S1, S2). The AUC of combined detection of the three indexes (*POSTN*, *PENK* and *CDC25A*) was 0.98 (Fig. 7D and Table 2). Taken together, our study demonstrated that the three candidate hub genes were significantly related to AR diagnosis.

**Table 2:** AUCs for predictors associated with AR

Gene	AUC	95% CI	*P* value
*POSTN*	0.85	0.663 to 1.037	0.0082
*PENK*	0.78	0.5727 to 0.9873	0.0343
*CDC25A*	0.77	0.5597 to 0.9803	0.0413
3 gene combinations	0.98	0.9295 to 1.031	0.0003

*AUC* area under the curve, *CI* confidence interval

## Discussion

Our study was based on nasal epithelial cells and Th2-enriched CD4+ cells from the peripheral blood of AR patients and nonallergic controls from the GEO database. We identified 997 n-DEGs (438 up-regulated and 559 down-regulated) from nasal epithelial cells and 165 t-DEGs (78 up-regulated and 87 down-regulated) from Th2-enriched CD4+ T cells. In addition, we identified 8 and 14 hub genes from PPI networks of the n-DEGs and t-DEGs that might be used as biomarkers in the pathogenesis of AR. Some of the hub genes have been studied in AR. *MMP9*, as a member of the matrix metalloproteinase family, has been demonstrated to act in a proinflammatory manner. The increased expression of *MMP-9* in allergic nasal mucosa contributes to the migration of inflammatory cells to the nasal mucosa of AR patients [22, 23]. *POSTN* encodes a secreted extracellular matrix protein that functions in tissue development and regeneration and is upregulated in AR [24]. *PTGDR2* is a prostaglandin D2 receptor that mediates the proinflammatory chemotaxis of eosinophils, basophils, and Th2 lymphocytes generated during allergic inflammation. *PTGDR2* can also be used as a Th2-related gene to evaluate the effect of AR immunotherapy [25]. A study on nasal tissue from individuals with allergic and nonallergic chronic rhinosinusitis highlighted a novel role for *CX3CR1* (i.e., *CX3CR1* may contribute to natural killer cell trafficking to the allergic upper airway) [26]. The production of CCL20 in human nasal epithelial cells may increase after stimulation dsRNA [27], and the upregulation of CCL20 expression may contribute to the recruitment and retention of effector T cells in allergic asthma [28]. *BGN*, one of the small leucine-rich repeat proteoglycans involved in tissue remodeling in inflammatory diseases, showed strong immunoreactivity in mild and severe persistent allergic nasal mucosa [29].

In some previous studies, bioinformatic analysis of mRNAs and lncRNAs in AR was performed. For instance, two studies focused on DEGs from the nasal epithelial cells of AR patients [9, 30]. Another study focused on DEGs and the transcription factor (TF)-miRNA network only in nasal epithelial cells and showed that miR-17-5P might act in SAR by targeting ATP binding cassette subfamily A member 1 (ABCA1) and CD69 [31]. Additionally, a study on DEGs in adult and pediatric nasal epithelial cells a found that AP-1 was associated with AR by regulating cystatin SN (CST1) and Charcot–Leyden crystal galectin (CLC) [32]. Circulating miR-125b, miR-16, miR-299-5p, miR-126, miR-206, and miR-133b in peripheral blood have been identified as predictive markers of allergic and asthmatic status [33]. A study based on Th2-enriched CD4+ T cells from peripheral blood samples revealed unique and common molecules that are likely to confer pathogenic features to Th2 cells in asthma and rhinitis [11]. These results together with the DEGs identified in different sources of samples suggest few intersecting genes obtained using different tissues or cells from different locations [34, 35]. Inherent gene expression due to potential confounders produced from highly expressed genes in different tissue or cell types might be ignored in an analysis of DEGs in a single source. Recently, some gene expression studies on asthma [36], musculoskeletal development [37], and nonalcoholic fatty liver disease [38] have reported approaches in which different tissues were combined.

In our study, MetaCore pathway map analysis showed that NETosis was the most significant differentially enriched pathway between AR patients and healthy controls. Neutrophil extracellular traps are the result of a unique form of cell death that is morphologically characterized by the loss of intracellular membranes before the integrity of the plasma membrane is compromised; this is called NETosis [39]. NETosis has been reported to be involved in asthmatic airway inflammation and chronic rhinosinusitis (CRS). A study on a mouse model of asthma showed that rhinovirus infection triggers dsDNA release associated with the inhibition of NETosis [40]. CRS displays variable degrees of eosinophilic and neutrophilic inflammation, with profound neutrophilic infiltration and activation in type 2 CRS with nasal polyps (CRSwNP) [41]. The underlying mechanisms of NETosis would be of considerable promise for AR patients. NF-κB-, AP-1- and MAPK-mediated proinflammatory cytokine production by eosinophils might play an important role in the nasal mucosa in AR. Similar to our study, a previous bioinformatic study found that AP-1 was associated with AR by regulating CST1 and CLC [32, 42]. IL-1 family cytokines (IL-1β, IL-18, and IL-33), IL-25, and TNF superfamilies can activate the NF-κB and AP-1 pathways to initiate the production of IL-5 and IL-13 in allergic airway inflammation [43]. However, the roles of the NF-κB, AP-1 and MAPK pathways in AR still need to be completely explained. Chronic inflammation is a key factor that leads to tissue remodeling [44]. Whereas the epithelial to mesenchymal transition (EMT) process and remodeling have been proven to be characteristic of chronic airway inflammatory diseases such as asthma and CRS, the presence/absence and extent of tissue remodeling in the upper airways in AR are still controversial [5, 45, 46]. We also found that cell adhesion (extracellular matrix remodeling) and TGF-β1 in fibrosis development are prominent significant pathways in the nasal epithelial cells of AR patients, suggesting that cell adhesion and tissue remodeling in the nasal mucosa of AR patients need to be explored in the future.

Although our study demonstrated that combination of multiple gene biomarkers had more accuracy to be diagnostic for AR, there are some major gaps exist in the AR biomarker space. In particular, our study is limited with the small sample size from the AR patients and healthy controls. It’s better to include the data contains the progression of AR before and after treatment. Besides our study, additional work is needed to explore the underlying mechanisms that gene biomarkers involved.

## Conclusion

In summary, our study aim to identify genes and pathways in the nasal epithelial cells and peripheral blood of AR patients through publicly available datasets. We identified the DEGs and analyzed their functions through GO analysis and PPI networks and identified the hub genes that might play crucial roles in the pathogenesis of AR. In addition, we validated the hub genes both by GEO database and RT-qPCR in nasal mucosa and in blood from AR patients and the healthy controls. Our study demonstrated that the increasing *POSTN* expression in nasal brushings, and the decreasing expressions of *CDC25A* and *PENK* in blood in AR or their combination could be used to help AR diagnosis (AUC = 0.98). This study provides new insights into the overall mechanism of AR from the nasal cavity to the blood, but further research is needed to develop novel therapeutic strategies to drug discovery along with medical approaches to clinical treatment of AR.

## Supplementary Information

**Additional file 1: Figure S1. **Flowchart of our study to identify potential gene markers in AR patients. **Table S1. **The raw mean Ct value of the nasal brush samples and the blood samples from AR patients and healthy controls. **Table S2. **The RNA concentration of the nasal brush samples and the blood samples from AR patients and healthy controls.

## Data Availability

All data generated or analyzed during this study are included in this published article.
