# Supplementary material for: Identification of gene biomarkers with expression profiles in patients with allergic rhinitis
Source: Allergy Asthma Clin Immunol. 2022 Mar 4;18:20. doi: 10.1186/s13223-022-00656-4 (PMC8897927; doi:10.1186/s13223-022-00656-4)
Supplement: Supplementary file 1 — Additional file 1: Figure S1. Flowchart of our study to identify potential gene markers in AR patients. Table S1. The raw mean Ct value of the nasal brush samples and the blood samples from AR patients and healthy controls. Table S2. The RNA concentration of the nasal brush samples and the blood samples from AR patients and healthy controls. [file 13223_2022_656_MOESM1_ESM.docx]

**Additional Figures**

**Figure S1.** Flowchart of our study to identify potential gene markers in AR patients. AR, allergic rhinitis; GEO, gene expression omnibus; FC, fold change; PPI, protein-protein interaction; n-DEGs, differentially expressed genes in nasal epithelial cells; t-DEGs, differentially expressed genes in blood; RT-qPCR, real-time quantitative PCR; ROC, receiver operating characteristic.

**
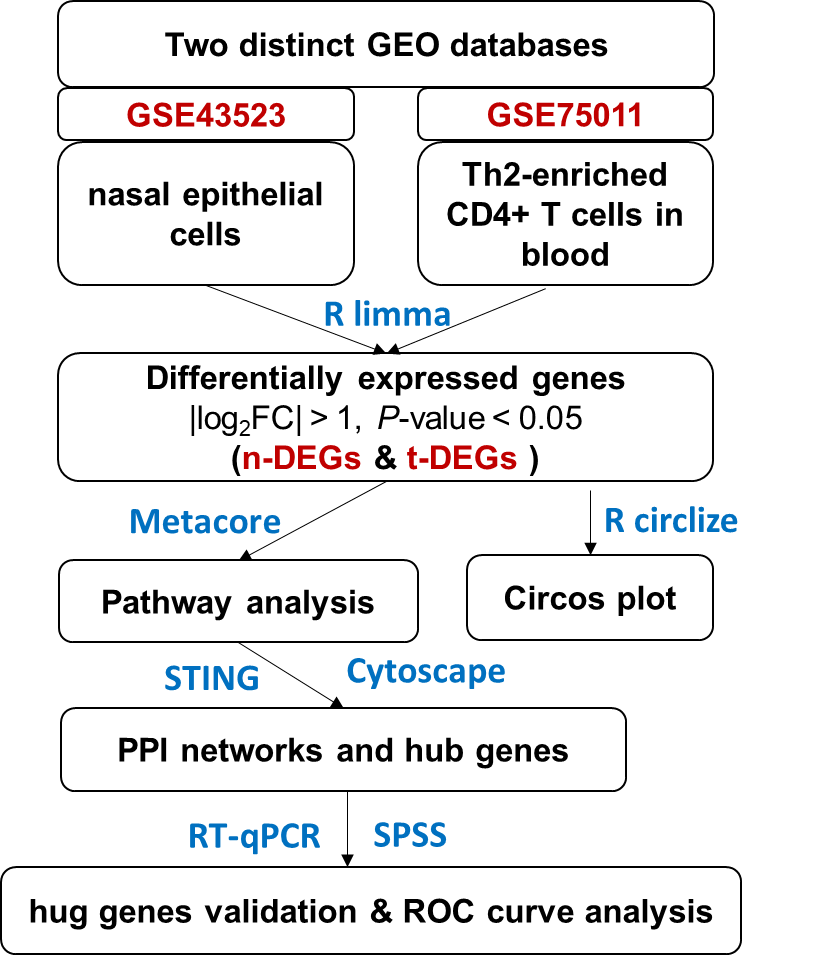
**

**Additional Tables**

**Table S1.** The raw mean Ct value of the nasal brush samples and the blood samples from AR patients and healthy controls.

| **Samples** | **nasal brush sample** | | **Blood sample** | | |
| --- | --- | --- | --- | --- | --- |
|  | ***GAPDH*** | ***POSTN*** | ***GAPDH*** | ***PENK*** | ***CDC25A*** |
| AR patient. 1 | 19.948 | 25.038 | 19.637 | 28.771 | 25.997 |
| AR patient. 2 | 20.888 | 27.468 | 18.946 | 28.103 | 26.149 |
| AR patient. 3 | 20.725 | 27.637 | 19.516 | 27.560 | 26.394 |
| AR patient. 4 | 21.202 | 26.303 | 19.329 | 27.529 | 25.772 |
| AR patient. 5 | 19.670 | 26.043 | 20.292 | 29.365 | 27.128 |
| AR patient. 6 | 20.629 | 29.574 | 20.599 | 30.347 | 27.719 |
| AR patient. 7 | 19.998 | 24.741 | 19.390 | 28.641 | 25.422 |
| AR patient. 8 | 21.080 | 26.466 | 20.349 | 30.411 | 26.298 |
| AR patient. 9 | 20.566 | 25.543 | 19.345 | 29.579 | 25.979 |
| AR patient. 10 | 20.312 | 26.134 | 18.249 | 26.789 | 24.574 |
| Control. 1 | 20.803 | 27.651 | 19.617 | 28.357 | 27.066 |
| Control. 2 | 20.408 | 31.659 | 20.422 | 28.819 | 27.095 |
| Control. 3 | 21.310 | 29.714 | 18.777 | 28.247 | 25.972 |
| Control. 4 | 20.397 | 27.009 | 19.595 | 27.200 | 26.867 |
| Control. 5 | 19.631 | 28.132 | 19.308 | 26.901 | 26.279 |
| Control. 6 | 20.679 | 29.474 | 20.243 | 28.425 | 27.272 |
| Control. 7 | 20.756 | 27.457 | 18.828 | 26.729 | 26.244 |
| Control. 8 | 21.392 | 27.430 | 19.989 | 29.305 | 28.020 |
| Control. 9 | 20.440 | 27.435 | 20.205 | 28.500 | 26.487 |
| Control. 10 | 19.970 | 27.917 | 19.516 | 27.401 | 25.888 |

**Table S2.** The RNA concentration of the nasal brush samples and the blood samples from AR patients and healthy controls.

| **Sample ID** | **Conc.** | **Unit** | **A260** | **A280** | **260/280** | **260/230** | **Sample type** |
| --- | --- | --- | --- | --- | --- | --- | --- |
| AR patient 1 | 152.3 | ng/µL | 3.809 | 1.905 | 2 | 2.27 | nasal brush |
| AR patient 2 | 139.6 | ng/µL | 3.491 | 1.795 | 1.95 | 2.38 | nasal brush |
| AR patient 3 | 135.4 | ng/µL | 3.385 | 1.698 | 1.99 | 2.28 | nasal brush |
| AR patient 4 | 136.6 | ng/µL | 3.416 | 1.713 | 1.99 | 2.52 | nasal brush |
| AR patient 5 | 135.9 | ng/µL | 3.398 | 1.68 | 2.02 | 1.93 | nasal brush |
| AR patient 6 | 123.1 | ng/µL | 3.077 | 1.535 | 2 | 2.42 | nasal brush |
| AR patient 7 | 118.4 | ng/µL | 2.961 | 1.456 | 2.03 | 2.03 | nasal brush |
| AR patient 8 | 147.8 | ng/µL | 3.694 | 1.889 | 1.96 | 2.48 | nasal brush |
| AR patient 9 | 142.2 | ng/µL | 3.555 | 1.8 | 1.97 | 2.24 | nasal brush |
| AR patient 10 | 172.3 | ng/µL | 4.306 | 2.138 | 2.01 | 2.41 | nasal brush |
| Control 1 | 150.9 | ng/µL | 3.771 | 1.873 | 2.01 | 2.07 | nasal brush |
| Control 2 | 152.8 | ng/µL | 3.821 | 1.873 | 2.04 | 2.39 | nasal brush |
| Control 3 | 128 | ng/µL | 3.2 | 1.67 | 1.92 | 2.43 | nasal brush |
| Control 4 | 171.7 | ng/µL | 4.292 | 2.16 | 1.99 | 2.34 | nasal brush |
| Control 5 | 161.4 | ng/µL | 4.034 | 2.015 | 2 | 2.43 | nasal brush |
| Control 6 | 122.7 | ng/µL | 3.067 | 1.58 | 1.94 | 2.4 | nasal brush |
| Control 7 | 110.5 | ng/µL | 2.763 | 1.378 | 2.01 | 2.47 | nasal brush |
| Control 8 | 138.2 | ng/µL | 3.455 | 1.694 | 2.04 | 2.44 | nasal brush |
| Control 9 | 174 | ng/µL | 4.349 | 2.165 | 2.01 | 2.45 | nasal brush |
| Control 10 | 189.3 | ng/µL | 4.733 | 2.441 | 1.94 | 2.49 | nasal brush |
| AR patient 1 | 497.7 | ng/µL | 12.44 | 6.512 | 1.91 | 2.5 | blood |
| AR patient 2 | 530.1 | ng/µL | 13.25 | 6.893 | 1.92 | 1.48 | blood |
| AR patient 3 | 567.7 | ng/µL | 14.19 | 7.407 | 1.91 | 2.47 | blood |
| AR patient 4 | 673.6 | ng/µL | 16.84 | 8.753 | 1.92 | 2.46 | blood |
| AR patient 5 | 646.7 | ng/µL | 16.17 | 8.266 | 1.96 | 2.39 | blood |
| AR patient 6 | 592.9 | ng/µL | 14.82 | 7.664 | 1.93 | 2.37 | blood |
| AR patient 7 | 467.3 | ng/µL | 11.68 | 5.893 | 1.98 | 2.56 | blood |
| AR patient 8 | 887.85 | ng/µL | 22.2 | 11.23 | 1.93 | 2.59 | blood |
| AR patient 9 | 725.4 | ng/µL | 18.13 | 9.4 | 1.93 | 2.43 | blood |
| AR patient 10 | 689.52 | ng/µL | 16.04 | 8.23 | 1.95 | 2.29 | blood |
| Control 1 | 711.2 | ng/µL | 17.78 | 8.55 | 2.08 | 0.91 | blood |
| Control 2 | 482 | ng/µL | 12.05 | 6.055 | 1.99 | 2.38 | blood |
| Control 3 | 512.5 | ng/µL | 12.81 | 6.6 | 1.94 | 2.3 | blood |
| Control 4 | 466.8 | ng/µL | 11.67 | 6.051 | 1.93 | 2.26 | blood |
| Control 5 | 768.9 | ng/µL | 19.22 | 9.42 | 2.04 | 2.39 | blood |
| Control 6 | 586.6 | ng/µL | 14.66 | 7.41 | 1.98 | 2.04 | blood |
| Control 7 | 574.1 | ng/µL | 14.35 | 7.29 | 1.97 | 2.57 | blood |
| Control 8 | 642.8 | ng/µL | 16.07 | 7.92 | 2.03 | 2.88 | blood |
| Control 9 | 760.12 | ng/µL | 19.01 | 9.61 | 1.98 | 2.36 | blood |
| Control 10 | 641.58 | ng/µL | 17.24 | 8.58 | 2.01 | 2.25 | blood |
